# Supplementary material for: Fifty years of change in the lower tree line in an arid coniferous forest in the Qilian Mountains, northwestern China
Source: PLoS One. 2023 Oct 12;18(10):e0292682. doi: 10.1371/journal.pone.0292682 (PMC10569624; doi:10.1371/journal.pone.0292682)
Supplement: Attachment — Submitted filename: Response to Reviewers.docx [file pone.0292682.s002.docx]

Dear Editor and Reviews:

Thank you for your comments on our manuscript titled “Fifty years of change in the lower tree line in the Qilian Mountains, northwestern China" (PONE-D-23-19267R1). The comments were invaluable for improving our manuscript and contributed important insights for our further research.

We have carefully addressed the comments and made corrections to the best of our abilities. We hope that our corrections meet with your full approval. Revised portions of the text are marked in the “track changes” function (MS Word) in the manuscript. The revised paper has been thoroughly checked by a native speaker for language and consistency. These changes do not influence the content and framework of the paper.

We uploaded our figure files to the Preflight Analysis and Conversion Engine (PACE) digital diagnostic tool, https://pacev2.apexcovantage.com/. and downloaded the processed images and uploaded them to the submission system.

Once again, thank you very much for your comments and suggestions. The main corrections in the paper and responses to the reviews and editor's comments are as follows:

Journal Requirements: Please review your reference list to ensure that it is complete and correct. If you have cited papers that have been retracted, please include the rationale for doing so in the manuscript text, or remove these references and replace them with relevant current references. Any changes to the reference list should be mentioned in the rebuttal letter that accompanies your revised manuscript. If you need to cite a retracted article, indicate the article’s retracted status in the References list and also include a citation and full reference for the retraction notice.

Response: We checked our references again and made corrections.

Additional Editor Comments:

The authors dealt with the comments reasonably. However, some uncertainty remains and should be mentioned in the abstract. For example, human activity is an important factor influencing the treeline migration and should be indicated in the abstract. In addtion, since the authors use a short period 1968-2018 with a decadal resolution for analysis. I will be subjected to uncertainties due to a small number of degree of freedom.

Response: Thank you for this suggestion. We added the content of the effects of human activities on the tree line in the abstract. Additionally, we strengthened the discussion of the uncertainty caused by a relatively short temporal resolution and a small number of degree of freedom.

Changes in abstract：

The change in the lower tree line was significantly correlated with the annual average temperature (℃) and annual precipitation (mm) and may be affected by human activities..

The part added in 4.3

*Picea crassifolia* Kom. grows slowly, so we chose ten years as the time scale for studying tree line change. Then, to explore the relationship between tree line change and meteorological factors, the correlation analysis was done at that time scale, too. Therefore, the amount of data is small, and analysis of the relationship between tree line changes and meteorological factors supported the discussion on the possibility of tree line changes only. In follow-up work, tree rings will be considered to more accurately establish the relationship between tree growth and climate change.
